# *Leptospira* Status in Sweden during the Past Century, Neglected and Re-Emerging?

**DOI:** 10.3390/microorganisms11081991

**Published:** 2023-08-02

**Authors:** Tanja M. Strand, Eva Olsson Engvall, Elina Lahti, Marika Hjertqvist, Åke Lundkvist

**Affiliations:** 1 National Veterinary Institute, SE-751 89 Uppsala, Sweden; 2 Zoonosis Science Center, Department of Medical Biochemistry and Microbiology, Uppsala University, SE-751 23 Uppsala, Sweden; 3 Department of Biomedical Sciences and Veterinary Public Health, Swedish University of Agricultural Sciences, SE-750 07 Uppsala, Sweden; 4 Department of Communicable Disease Control and Health Protection, Public Health Agency of Sweden, SE-171 82 Solna, Sweden

**Keywords:** *Leptospira*, surveillance, zoonosis, microscopic agglutination test, One Health

## Abstract

We compiled data on notified cases of leptospirosis in animals and humans in Sweden. Published studies on leptospirosis in humans and animals from the beginning of the 20th century onwards are summarized. During the Second World War, hundreds of leptospirosis cases in humans were reported in Sweden, but since then, there have been only a few severe cases. Surveillance of leptospirosis in domestic animals demonstrates that the pathogen is still occurring. The occurrence of *Leptospira* in humans and animals in the other Nordic countries resembles that in Sweden. Leptospirosis is an underdiagnosed and underreported disease globally, both in animals and humans, partly due to the lack of simple, rapid diagnostic tools but possibly also due to the lack of awareness among physicians, veterinarians and nurses. Traditionally, leptospirosis has been mostly diagnosed by serology, but development of molecular methodshas improved the capability for correct diagnosis. As of today, leptospirosis is regarded as a relatively uncommon disease in the Nordic countries, but in some other countries, it is considered a neglected zoonosis or a (re-)emerging disease that may become more common in the future. Possible factors that could contribute to an increase in incidence are discussed in this review. Active surveillance of humans and domestic and wild animals and stringent rodent control in society and animal farms are of outmost importance for prevention.

## 1. Introduction 

### 1.1. Leptospirosis—What Is the Problem and Where? 

Leptospirosis is a potentially fatal bacterial zoonosis. Infection is widespread across the globe, with an estimated number of one million human cases per year [1]. Leptospirosis is most common in areas with a tropical climate, and the incidence in humans is highest in the Caribbean region, Central and South America and Southeast Asia and Oceania [2]. The incidence varies between regions. In the European Union, between 439 and 1049 confirmed cases in humans were reported annually between 2010 and 2021 [3], and the numbers fluctuate.

*Leptospira* spp. may affect a wide variety of animal species, both wild and domestic [4]. In addition to the suffering the infection causes to the affected animal or human, leptospirosis in food-producing animals leads to considerable economic losses, as well as to a risk of transmission of the pathogen to humans. Estimating the impact of leptospirosis on livestock production is challenging, but the highest economic losses are encountered in areas with a tropical climate [2,5].

In this study, we aimed to describe the epidemiology, diagnostics, clinical characteristics and surveillance of *Leptospira* and leptospirosis in Sweden and the other Nordic countries between 1922 and 2022 by compiling data from published studies and databases at the national public health and veterinary institutes of the Nordic countries.

### 1.2. Leptospira Classification

Leptospires are slow-growing, fastidious organisms that need specific culture media and conditions for growth. This has complicated traditional bacteriological methods for detection, characterisation and classification. To circumvent the problem, serological classification based on surface-exposed lipopolysaccharides has been used [4], identifying more than 300 serovars [6]. Antigenically related serovars have been grouped into serogroups [4,7]. Recently, the taxonomy of leptospires has been revised by genotypic classification. Currently, the *Leptospira* genus is suggested to be divided into two clades, P and S, each further divided into two subclades. Subclade P1 contains the *Leptospira* spp. formerly described as constituting the pathogen group, P2 contains the intermediate group and the species in the S clade are the saprophytic groups [8]. However, for clinicians, microbiologists and epidemiologists, the nomenclature with serovars will continue to be used for practical reasons [7,9].

### 1.3. Hosts, Reservoirs and Transmission of Leptospira spp.

*Leptospira* spp. have been isolated from many animal species. Wild rodents, like the brown rat (*Rattus norvegicus*) and the house mouse (*Mus musculus*), but also insectivores, such as the European hedgehog (*Erinaceus europaeus*), are the primary reservoirs or so-called maintenance hosts for *Leptospira* spp. The brown rat is considered the main reservoir for the serovar Icterohemorrhagiae. *Leptospira* spp. reside in the kidneys of these reservoir animals, which are, in general, asymptomatic. The leptospires are shed in the urine, thus contaminating soil and water [4]. The transmission to accidental or secondary hosts (i.e., to humans and domestic and other animals) occurs via contaminated soil or water or by direct contact with urine or organs from infected animals [4,10,11]. The leptospires may also enter the body via cuts and abrasions in the skin or via mucous membranes, whereafter they reach the circulation and spread to different tissues and organs [12].

All pathogenic *Leptospira* serovars are potentially able to infect all animal species, although some serovars are more adapted to certain animal species. Examples of such host/reservoir relationships in domestic animals are *L*. Hardjoe in cattle and *L*. Canicola in dogs. Pigs could be reservoirs of *Leptospira* serovars Pomona and Tarrasovi [13].

## 2. Material and Methods

We searched scientific articles and compiled data from case-based and active surveillance retrieved from national authorities. Published studies on historical *Leptospira* and leptospirosis cases, observations and experiments were retrieved, mainly from the reference lists of previous publications. Data on reported leptospirosis cases in humans in Sweden were retrieved from the reporting system of the Public Health Agency of Sweden. For 1993–2003, data were retrieved from the archives of the Swedish Institute for Infectious Disease Control. Data from prior to 1993 were retrieved from Linglöf [14]. Data for 2007–2019 on animals were obtained from archives of the National Veterinary Institute (SVA), Uppsala, Sweden. Information about the notified cases (or findings) of leptospirosis in animals and humans was also obtained from Denmark, Finland, Iceland and Norway.

### 2.1. Culture, Microscopy and Molecular Methods for Laboratory Analyses

Isolation of leptospires from clinical material is difficult and only feasible at reference laboratories with adequate equipment and expertise. In older studies from the 1930s and 1940s, susceptible experimental animals (i.e., guinea pigs or hamsters) were inoculated with sample material in order to enhance the possibility of isolation of leptospires by culture in special liquid media [5,15,16].

Using dark-field microscopy, leptospires may be detected in body fluids, like blood, urine and cerebrospinal fluid. To improve the specificity of the method, staining with specific immunofluorescent antibodies can been used [5,7]. The leptospires may also be visualized; e.g., by silver staining (Levaditi) in histopathological specimens [7].

Molecular methods have been developed for the specific detection of leptospiral DNA in clinical material. Several PCR (Polymerase Chain Reaction) assays have been described and are increasingly applied for detection of *Leptospira* [17].

### 2.2. Serological Methods

#### Microscopic Agglutination Test (MAT)

Partly due to problems with other techniques, laboratory diagnosis of leptospirosis has been most commonly based on serology. The MAT is universally considered the reference or “golden standard” method. In the MAT, titrated patient (human or animal) serum is mixed with suspensions of live *Leptospira* bacteria of different serovars and examined microscopically for agglutination. The highest dilution of serum at which 50% of the leptospires have agglutinated is regarded as the endpoint (titre) for a positive sample. Usually, a titre of ≥1:100 is used as a threshold for *Leptospira* infection or as evidence of past exposure [5,15,16]. However, the set threshold has varied over time and between laboratories and surveys. Cross-reactions between serogroups and serovars occur. Animal sera are usually screened against a couple of serogroups or serovars depending on animal species, epidemiology and specific requests, such as import or export of animals. If the screening results in a titre ≥ 1:100, the actual serum is tested against a wider range of serovars and the serovar with agglutination in the highest dilution is regarded the causative agent. Criteria for how to perform the test and interpret the results have been established [5,15,16].

### 2.3. Other Serological Tests

The MAT is serogroup/serovar-specific but cumbersome to perform and requires a set of *Leptospira* strains of different serovars for testing patient or animal sera. This is usually only possible for a limited number of reference laboratories to maintain. Therefore, many other serological tests have been developed. These are mainly used for testing human samples; e.g., the macroscopic slide agglutination test, the complement fixation test, dipstick tests, latex agglutination tests and ELISA (enzyme-linked immunosorbent assay) tests [5,7,17]. Many of these tests are commercially available, easy to perform and relatively rapid. ELISA assays are widely applied and can detect IgM antibodies at the early stages of the infection. In contrast to the MAT, many other serological tests are only genus-specific. Evaluation of the non-MAT serological tests in terms of sensitivity has been discussed [17]. Nevertheless, there seems to be a consensus that, for a definite diagnosis of leptospirosis, positive test results should be confirmed by the MAT [5,17].

### 2.4. Techniques Used for Laboratory Analyses in Sweden

The diagnostic techniques applied have varied over time. In the first Swedish studies of leptospirosis in humans and animals in the 1930s, laboratory diagnosis was performed at the Swedish Bacteriological Laboratory (SBL, currently the Public Health Agency of Sweden), Solna, Sweden, using dark-field microscopy, guinea pig inoculation and the MAT [18,19,20]. In the early days, it was only possible to identify antibodies to the serovar Icterohemorrhagiae. A few years later, an *L*. Canicola strain was added to the serological diagnostics [21,22]. Later, other serological tests were introduced at the Public Health Agency of Sweden for testing human samples; i.e., the commercial macroscopic slide agglutination test (from Institut Pasteur, Paris, France, and later from Bio-Rad, Berkeley, CA, USA). From 2012 onwards, a commercial IgM-ELISA test has been used at the Public Health Agency of Sweden. Following an agreement with the Statens Serum Institut (SSI, Copenhagen, Denmark), human sera with positive reactions in the ELISA (and earlier in the macroscopic slide agglutination test) have been sent to the SSI for confirmation and serovar identification with the MAT.

For testing animal samples, the National Veterinary Institute (SVA), Uppsala, Sweden, has, since 1946, performed routine screening using the MAT with live antigens. In the beginning, only a few serovars were included, but over time, more serovars have been added to represent all relevant serogroups. As of today, up to about 20 serogroups or serovars can be tested with the MAT at the SVA. The threshold for a positive reaction was, until the 1990s, set at 1:300 but, currently, a titre of ≥1:100 is applied as the threshold. Since 2015, PCR [23] has been increasingly used for detection of *Leptospira* DNA at the SVA. Histological methods have also been applied. 

A summary of methods used for diagnosis of leptospirosis in animals and humans in Sweden is shown in Table 1. Comprehensive technical information about the different tests can be found in [5,7,15,16,23].

## 3. Clinical Manifestations and Epidemiology 

### 3.1. Leptospirosis in Humans

*Leptospira* spp. can cause a mild, self-limited febrile illness and, more rarely, a severe disease characterized by dysfunction of multiple organs, including the liver, kidneys, lungs and brain [4]. The classical severe leptospirosis, also called Weil’s disease, is a critical condition presented by jaundice, renal failure, haemorrhages and myocarditis with arrhythmias [5].

Leptospirosis in humans is often associated with certain occupations or leisure time activities involving agriculture, contact with wild or domestic animals or outdoor activities and environmental risks, such as heavy rainfalls and flooding [24].

#### 3.1.1. History

The first cases of leptospirosis in humans in Sweden and in the other Nordic countries were associated with exposure to rats.

The first laboratory-confirmed human case of leptospirosis in Sweden was reported in 1922 in a person residing in Lackalänge in the southernmost region of Sweden, Skåne [25] (Table 2). However, already in 1918, a female fatal case with both clinical and post mortem findings consistent with leptospirosis was retrospectively identified [25]. However, the disease seems to have occurred already in the late 20th century in the Nordic countries. Although the discovery of the clinical disease was first documented by Weil in 1886 in Germany [26], Runeberg documented two cases with icteric fever in Finland as early as in 1878 [27]. The similarity of these two cases with Weil’s disease was discovered later [27]. The first leptospirosis case in Norway was reported in 1926 in a 42-year-old man who had fallen ill a year earlier [28]. In Denmark, the first verified human cases of leptospirosis were observed in 1934 [11].

Between 1918 and 1939, 114 cases of leptospirosis were documented in Sweden [18,21,25]. Out of these 114 patients, epidemiological data were available for 93 of them [21]. Rats were suspected as the source of the infection for 62 (67.7%) of these 93 patients. In the neighbouring countries, rats were linked to the infection of many cases as well. Out of 35 cases in Norway in the years from 1936 to 1940 [28], epidemiological data were obtained for 30 cases. For 22 (73.3%) of these 30 patients, contact with or the presence of rats were documented. In patient material from Finland from 1943 to 1952, 8 of 33 (24.2%) cases reported having seen plenty of rats and mice at their work place or at home [29].

#### 3.1.2. Leptospirosis Peaks and Drops in Numbers

**Figure 1:**
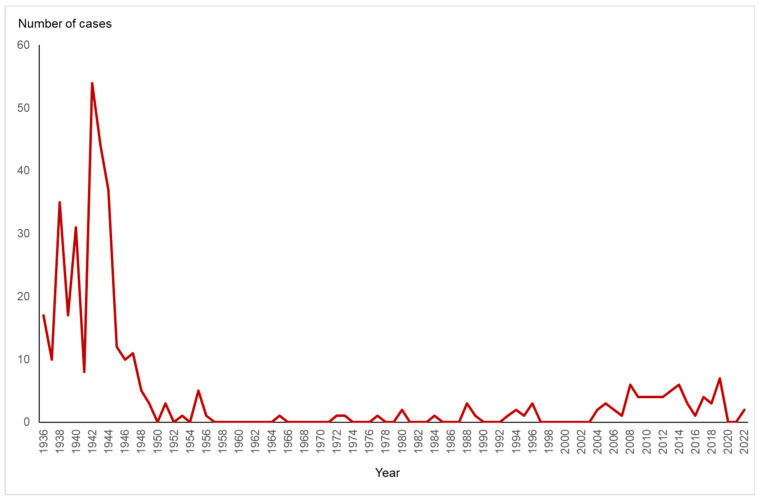
Number of reported human leptospirosis cases in Sweden from 1936 to 2022 (for data availability, see the Materials and Methods). Up to 1980, the numbers were based on clinical reports and, for most of the cases, it is unknown if the diagnosis was laboratory-confirmed or not. From 1998 to 2003, leptospirosis was not notifiable. From 2004 onwards, it has been mandatory for the treating physician to report laboratory-confirmed cases.

In Sweden, large peaks were observed between 1938 and 1944, with 226 clinically diagnosed leptospirosis cases reported (Figure 1). In Denmark, many human cases of leptospirosis were reported during World War II (particularly in 1943). Most of these patients lived in rural areas. A rise in the population of house mice (*Mus musculus*) was observed during the same time period in Denmark [11]. In Denmark, the house mouse is the maintenance host of the serovar Sejroe, and most human leptospirosis cases during the peak were caused by serovars Sejroe and Saxkoebing. Peaks in the incidence of leptospirosis were observed in the Netherlands in 1932 and 1941 [30]. Goris considered that the first peak coincided with the global economic depression and compulsory reporting of leptospirosis and the second peak with World War II [30].

After the rise in the number of patients during the 1940s, the number of reported cases has decreased. In Sweden, since 1948, fewer than 10 cases have been reported annually (Figure 1). The same trend is seen in Denmark [11,31,32,33]. Better household conditions and effective rat extermination are considered the main reasons for the decrease in the number of reported cases of leptospirosis [34]. The reduction in the Danish cases was considered a result of higher living standards but also of increased mechanization of agricultural and industrial operations [11].

**Table 2:** Published studies of human cases of leptospirosis in Sweden (1918–1972) ^1^.

Year (Reference)	Type of Study/Year	Result	Methods
1923 [25]	Case study Patient one, 1918 Patient two, 1922		Part one: clinical diagnostics Part two: guinea pig inoculation
1934 [20]	Summary of seven cases between 1922 and 1934	Same cases as Malmgren 1936 [18] and Malmgren 1941 [21])	MAT
1936 [18]	Summary of 22 cases between 1922 and 1935		MAT
	Surveillance of blood material from “fever” patient	7/1104 positive	MAT
1941 [21]	Description of 114 cases, 1918–1939	Both clinical description and epidemiology. Thirty-six worked as farmers or in stables. Cases year round but mainly in August	Guinea pig inoculation, MAT, complement fixation test
1972 [34]	Case study, one patient, rats at work, 1972	L. Ictero (1:300, 1:1000)	Agglutination test against killed spirochetes, confirmed by MAT

^1^ Since 1972, there have been no published articles about human leptospirosis in Sweden.

#### 3.1.3. Leptospirosis in 2000–2022

Since 2004, leptospirosis has been notifiable in Sweden according to the Communicable Disease Act (SFS 2004:168 with the amendments of SFS 2022:217 [35]). Up to 2022, zero to eight cases were reported annually. Almost all of the reported cases originated from infections abroad, many of them while visiting tropical areas and performing various water sports. During this time period, five notified cases probably acquired the infection in Sweden; two had documented contact with rats prior to the onset of symptoms. For the remaining three, the source of infection is more unclear.

In Denmark, between 3 and 23 cases have been reported annually since 2010. A higher incidence was noted in 2016 to 2018 than in 2010 to 2014. About half of the cases were imported. Of the domestic cases reported, most were considered to have contracted the infection following exposure to freshwater, including some occupational cases infected via contact with sewage, sludge and fish farm water. About a third of the domestic cases reported contacts with rats (https://en.ssi.dk/news/epi-news/2019/no-4-5—2019, accessed on 3 November 2021). Trends in human leptospirosis in Denmark from 1980 to 2021 have been studied and described in two articles [31,33].

In Norway, leptospirosis has not been a notifiable disease since 1994. However, since the Norwegian Institute of Public Health is the only laboratory where *Leptospira* diagnostics is performed in Norway, some data are still available, although samples may be sent abroad for analysis. Usually, one or two cases are detected every year. All the cases are considered to have been infected abroad. There are no signs of domestic transmissions (personal communication).

In Finland, the yearly number of notified cases varied between zero and eight from 2010 to 2021 (personal communication).

In Iceland, leptospirosis in humans is notifiable, but there have never been any cases reported, neither domestic nor imported (personal communication).

#### 3.1.4. Seasonality

In temperate regions, leptospirosis in humans is seasonal, with a peak incidence in summer or fall [7]. Earlier, in Sweden, based on data for 112 cases in 1918–1939, leptospirosis cases were observed throughout the year but mainly in July–October, with a peak in August [21]. Currently, few domestic cases are notified, thus complicating the assessments of seasonality. The same applies for Norway, Denmark and Finland, with year-round cases with a peak in late summer and/or autumn [28,29,32,33]. Notably, for Denmark, infections caused by serovars other than *L.* Icterohaemorrhagie were more evenly distributed across the year [32,33].

### 3.2. Leptospirosis in Animals

Like in humans, the clinical picture of leptospirosis in domestic animals ranges from a mild, subclinical infection to an acute or lethal infection. A chronic state, including shedding of the bacteria in the urine, may develop after the acute phase. The infection in animals is therefore impactful both as a threat to humans and as a clinical illness in animals, often with economic losses. Leptospirosis was first described in animals in 1850, when a disease called ”Stuttgarter Hundeseuche” was observed in dogs in Germany. Since then, leptospirosis has been described in most mammalian species all over the world [13]. In domestic animals, similarly to humans, a wide range of clinical signs can be seen: fever, malaise, diarrhoea, haemorrhages, renal failure, jaundice, abortion, stillbirth, agalactia and respiratory and neurological signs. Some manifestations are typically found in certain animal species; e.g., ”moon blindness” in horses, ”milk drop syndrome” in dairy cows and ”abortion storms” in cattle and pig herds. Historically, clinical leptospirosis in dogs has been characterised as either a fulminant, acute form (Stuttgarter Hundeseuche) or a less severe or subclinical form with shedding of leptospires via urine.

Although clinical signs of leptospirosis have not been recognised in wild mammals, antibodies against *Leptospira* spp. have been found in ungulates, like roe deer (*Capreolus capreolus*), and carnivores, like the red fox (*Vulpes vulpes*) [11].

Until 2005, leptospirosis in animals was on List B of diseases notifiable to the World Organisation for Animal Health (WOAH, founded as the OIE) [36], and there was an obligation to test animals subject to trade with the MAT as the standard serological test [16]. Although leptospirosis is no longer on the WOAH list, most countries still require testing of animals before import or export. In Sweden, many animals are tested for this reason (or others) [37]. 

#### 3.2.1. *Leptospira* in Swedish Animals

Findings of *Leptospira* spp. and/or antibodies to *Leptospira* in wild and domestic animals in Sweden have been reported since the 1930s. However, the frequency of publications has varied over time. In the years 1933 to 1956, several reports were published, followed by a long period with hardly any reports. A renewed interest in *Leptospira* in Swedish animals has emerged, which has resulted in more publications since 2006 (Table 3).

#### 3.2.2. Wild Rodents

Wild rodents were among the first animals to be studied. Using dark-field microscopy, guinea pig inoculation and the MAT, Olin [19,20] and Malmgren [21] could demonstrate the presence of *L*. Icterohemorrhagiae in up to 43% of brown rats but not in black rats or house mice [19,20,21].

More recently, Backhans et al. [38] reported findings of *Leptospira* DNA using PCR in wild rodents. In 2015, antibodies to *L*. Icterohemorrhagiae and to a Swedish *Leptospira* strain (Mouse 2A) were found in brown rats captured in larger Swedish cities [39]. Later, Strand et al. [40] also found brown rats positive for *Leptospira* using PCR in Swedish cities.

**Table 3:** Published studies on leptospirosis and/or findings of *Leptospira* spp. in Swedish animals (1933–2015).

Year (Reference)	Number of Positive/Number of Tested Animals (%) (Positive for *Leptospira* Icterohemorrhagia If Nothing Else Indicated)	Methods (MAT Serovars ^1^)
1933 [19]	Brown rat: 3/7	Guinea pig inoculation
1934 [20]	Brown rat: 6/92 (6.5%) Black rat: 0/11	Guinea pig inoculation
1937 [41]	Dog: 8/8 Farmed fox: 12/12	Microscopy, guinea pig inoculation, Levaditi silver staining
1941 [21]	Brown rat: 217/583 (37%) House mouse: 0/60 (0%)	Microscopy, guinea pig inoculation, MAT (serovar Ictero)
1941 [22]	Dog: 57/616 ^2^ (9.25%) Horse: 31/31 ^3^ (100%); 0/10 ^4^ Farmed fox: positive cases found (numbers not specified) Farmed mink: 3/57 (5%)	Microscopy, guinea pig inoculation, MAT (serovars Can and Ictero)
1956 [42]	Dog: 125/4450 ^5^ (2.8%) Horse: 75/588 ^5^ (11.4%) Pig: 42/1000 ^5^ (4.2%) Cattle: 58/555 ^5^ (10.5%) Farmed fox: 9/79 (11.4%) Farmed mink: 0/6	MAT (serovars Bat, Can, Gripp, Ictero, Pom, Sej)
1985 [43]	Horse: 44/89 ^6^ (49%), *L.* Bratislava Pig: 21/116 ^5^ (18.1%), *L.* Bratislava	MAT (serovars Brat, Hyos, Pom; horses at retest: Bat, Brat, Can, Hyos, Ictero, Gripp, Pom, Sej)
2006 [44]	Pig: 13/39 ^7^ (34%), *L.* Bratislava	MAT (serovars Brat, Gripp, Ictero, Pom, Tar)
2009 [45]	Horse: 335/2017 ^4^ (16.6%), *L.* Bratislava	MAT (serovars Brat, Gripp, Ictero, Pom, Sej)
2010 [46]	Wolf: 8/95 (8.4%) ^8^	MAT (serovars Can and Ictero)
2010 [47]	Case study: 1 dog, *L.* strain Mouse 2A	MAT (serovars Brat, Can, Gripp, Ictero, Pom, Sej, strain Mouse 2A)
2011 [48]	Cattle: 6/610 ^5^ (1%), strain Mus 2A	MAT (serovars Can, Gripp, Ictero, Sej, strain Mouse 2A)
2012 [49]	Pig: 31/386 ^5^ (8.0%), *L.* strain Mouse 2A	MAT (serovars Brat, Gripp, Ictero, Pom, Tar, strain Mouse 2A)
2012 [50]	Wild boar: 9/386 (2.3%), *L.* Bratislava	MAT (serovars Brat, Gripp, Ictero, Pom, Tar, strain Mouse 2A)
2013 [38]	Brown rat: 1/51 (2%) House mouse: 6/68 (9%) Yellow necked mouse: 1/5 Water vole: 1/1	PCR, sequencing
2015 [39]	Brown rat: 4/30 (13.3%)	MAT (serovars Ballum, Brat, Grippo, Ictero, strain Mouse 2A)

^1^ Serovars tested with the MAT. Explanation of abbreviations: Bat = Bataviae, Brat = Bratislava, Can = Canicola, Gripp = Grippotyphosa, Ictero= Icterohemorrhagiae, Pom = Pomona, Sej = Sejroe, Tar = Tarassovi. ^2^ Some dogs with leptospirosis symptoms. ^3^ Horses from a stable with suspected leptospirosis. ^4^ Horses with no suspicion of leptospirosis. ^5^ Healthy animals. ^6^ Some of the horses were from a stable with suspected leptospirosis. ^7^ Breeding pigs from herd with fertility problems. ^8^ Joint Swedish–Norwegian population of healthy wolves.

#### 3.2.3. Dogs

*Leptospira* was first observed in Swedish dogs in 1937 [41] (Table 3). In autopsy material from eight dogs with suspected ”Weil’s disease”, leptospires could be demonstrated with various techniques, such as using the Levaditi silver impregnation method for identification of histological findings. 

In two serological studies of leptospirosis in dogs, 9.25% and 2.8% of dogs, respectively, were positive in the MAT to *L*. Icterohemorrhagiae [22,42]. In the first study, some dogs with clinical symptoms were included and a titre of ≥1:100 was regarded as positive. In the latter study, only healthy dogs were tested and a higher threshold (≥1:300) was applied.

In a case study in 2010, a Swedish dog was diagnosed with leptospirosis based on clinical findings and high titres for the Swedish strain ”Mouse 2A”, characterised as *L*. Istrica/Sejroe-like [47]. The dog had not travelled outside Sweden and was thus classified as a domestic case of leptospirosis.

In a recent seroprevalence study of *Leptospira* in healthy Swedish dogs, 27/369 (7.3%) had MAT titres ≥ 1:50, indicating previous exposure to *Leptospira* Interrogans serovars [51].

Two asymptomatic dogs from Colombia, kept in quarantine in Sweden in 1971, were found positive with high MAT titres for *L*. Canicola. From the dog with the highest titre (1: 51,200), *L.* Canicola was isolated from the urine by culture [52].

#### 3.2.4. Horses

Nordström [22] studied the clinical course of leptospirosis in horses experimentally and naturally infected with *L*. Icterohemorrhagiae. Serological investigation of horses from a stable with suspected leptospirosis cases resulted in all horses (*n* = 31) being positive, whereas horses from a stable with no clinical suspicions were all negative.

In three other studies, proportions of seropositive animals varied depending on clinical status, serovar and the applied cut-off for positivity. In the studies by von Wendt [42] and Båverud et al. [45], asymptomatic horses were tested but different thresholds for positivity were applied (≥1:600 and ≥1:100, respectively). Still, fairly similar results were obtained: 11.4% and 8.3% of horses, respectively, were positive for *L*. Icterohemorrhagiae [42,45]. In a study by Sandstedt and Engvall [43], horses from a stable with suspected leptospirosis were included, and a higher proportion of horses with antibodies to the serovar Bratislava was found (49%) compared to the 16.6% in the study by Båverud et al. [45]. In both studies, other serovars were tested, but very few animals with only low titres were found.

#### 3.2.5. Pigs

In the first study on Swedish pigs, von Wendt found that 4.2% of the pigs tested were positive for *L.* Icterohemorrhagiae, with titres ≥ 1:400. A small number of pigs had lower titres for other serovars as well [42]. At the same time, other studies on pigs with clinical signs of leptospirosis were performed and referred to as personal communication by von Wendt. In the unpublished studies, high titres for *L*. Icterohemorrhagiae were found along with spirochetes in the urine and autopsy material from kidneys [42].

In the 1980s, *L*. Bratislava was reported associated with disease and reproductive problems in pigs in the UK and Ireland [53,54]. In Sweden, 18.1% of healthy pigs [43] and 34% of breeding pigs in a herd with fertility problems [44] were positive for *L*. Bratislava but not for other serovars. Among outdoor reared pigs, 8% had antibodies to the Swedish strain Mouse 2A, 3.9% to *L*. Bratislava and 1% to *L*. Icterohemorrhagiae [49].

#### 3.2.6. Cattle 

In a ”general study” (as expressed by von Wendt (1956) [42]) of samples from healthy animals, 10.5% had MAT antibodies to *L*. Icterohemorrhagiae and 2.9% to *L*. Pomona.

A more recent Swedish study of dairy cattle showed that 1% of the animals were seropositive and only to *Leptospira* strain Mouse 2A [48].

#### 3.2.7. Farmed Fur Animals and Wild Wolves/Boars

Leptospires were demonstrated in farmed fur animals by Rubarth [41] and Nordström [22], and von Wendt [42] reported that 11.4% of farmed foxes were positive for *L*. Icterohemorrhagiae.

Among free-ranging wild animals, 8.4% of wolves were positive for *L*. Icterohemorrhagiae but they were all negative for the serovar Canicola [46]. A small proportion of wild boars, 2.3%, were positive for *L*. Bratislava, while 0.8% were positive for *L*. Icterohemorrhagiae, and all of them were negative for the other serovars [50].

#### 3.2.8. Surveillance in Swedish Animals

Leptospirosis has been a notifiable disease for Swedish animals for many years. The basis for notification was specified in 2004. According to the present legislation (SJVFS 2021:10 [55]), leptospirosis is a notifiable disease upon laboratory confirmation for all animal species concerned. In 2021, the notification requirements for serological results were changed. Previously, findings of single serologically positive samples were notifiable; in 2021 legislation, two serologically positive results were required for notification based on serology, but this change was reverted back to single serological positive reactions being notifiable.

Passive surveillance with animals involves mandatory case reporting of laboratory-confirmed cases. Animals sampled for export and in breeding centres add to the passive surveillance. Active surveillance is carried out with cattle and pigs [37]. In cattle, serum and bulk milk samples are selected using systematic random sampling and the samples are tested for antibodies to *L*. Hardjo. The diagnostic test used for *L*. Hardjo is an indirect ELISA (PrioCHECK^®^ *L*. Hardjo, Ab Strip Kit, Prionics, Thermo Fisher Scientific, Lelystad, The Netherlands) for both serum and bulk milk samples. Positive serum samples are further tested with the MAT using a cut-off ≥100. If the serological result is positive or doubtful, an investigation is carried out in the herd and additional samples are taken.

Serum samples from pigs are taken at abattoirs using random sampling. The pig samples are tested for antibodies to *L.* Pomona with the MAT using the same cut-off as for cattle samples.

Between 1994 and 2006, sampling and testing for antibodies to serovars Hardjo and Pomona in cattle and pigs, respectively, were performed each year and, after 2006, every third year. The commercial cattle and pig populations in Sweden are considered free from *L*. Hardjo and *L*. Pomona based on the results from this surveillance system.

Surveillance with other animal species, including dogs and horses, is passive only.

The reasons for testing other animals (other than cattle and pigs) include suspicion of clinical disease and sampling for export/import requirements. Most samples are tested with the MAT at the SVA (cut-off for positivity ≥1:100), and different serovars are included depending on the animal species and reasons for testing. Samples are sometimes sent abroad and it is not known what test method is used or if positive results are being reported correctly [37].

Serovars generally included in tests of dog and horse sera, together with the distribution of positive samples in 2007–2019, are shown in Table 4.

The highest titres found (1:800–1:6400) were for the serovars Bratislava, Icterohaemorrhagiae and Mus 2A (Sejroe) in samples from both dogs and horses. For the other serovars, titres ranged from 1:100 to 1:400 with one exception: one dog had a titre of 1:800 for Saxkoebing. 

#### 3.2.9. Summary of Selected Articles from the Other Nordic Countries

Like in Sweden, *Leptospira* spp. and leptospirosis in animals have been reported to varying degrees in Denmark, Finland and Norway. In these countries, a high frequency has been found for *L*. Icterohemorrhagiae in brown rats, from 33% to over 50% depending on which method is used and the age of the rats [28,56,57,58]. Interestingly, in a study from the Faroe Islands (a territory of Denmark), all sampled brown rats (*n* = 95) were negative when tested by PCR for *L. interrogans* [59]. Also, other small rodents/mammals have been investigated. In Denmark, several serovars have been identified in small rodents, shrews, hedgehogs and bats; e.g., the *Leptospira* serovars Bratislava, Grippotyphosa, Icterohemorrhagiae, Poi, Pomona, Sejroe and Saxkoebing [11]. In Finland, Salminen [27] examined different small rodents and shrews and found serovars Sejroe, Bataviae and Poi.

In domestic animals, leptospirosis was first reported in Danish dogs, in which the infection was described as a common disease from 1938 to 1939 and was mainly caused by the serovar Canicola, as well as *L.* Icterohemorrhagiae [60,61]. Serological studies from 1959 to 1960 showed that 12.2% of dogs from the Copenhagen area and 23.3% from the provinces had antibodies to one or several *Leptospira* serovars: Sejroe, Bratislava, Canicola, Icterohemorrhagiae, Poi, Pomona and Bataviae (in order of decreasing frequency) [62]. In a Norwegian study, 6.5% of dogs with icterus and uraemia tested positive for leptospirosis [63]. Later, according to Sunde et al. [64], leptospirosis was suspected in several Norwegian dogs and was in two cases verified by microscopy and MAT serology, which showed high titres for *L*. Canicola.

In Finland, 18.9% (of 37) dog sera tested with the MAT were positive for *Leptospira* serovars Icterohemorrhagiae, Bataviae and Canicola in a study by Salminen [27]. In 1986, an outbreak with leptospirosis in puppies in a kennel was described [65]. At autopsy, spirochetes were found in kidney tissue with silver staining in one of the puppies. Antibodies to *L*. Sejroe were detected in both ill and asymptomatic puppies [65].

Leptospirosis in farm animals has also been studied in the Nordic countries. In Denmark, extensive studies on leptospirosis in cattle have been performed by Borg-Petersen and Fennestad [66,67]. In Finland, Salminen [27] found antibodies to *Leptospira* in only 3% of the 303 tested bovines. In a serological study from 1975 to 1985, a very low seroprevalence was demonstrated in the cattle population: 0.13% of 5984 samples were positive for the serovars Icterohemorrhagiae and/or Sejroe [68].

Studies of Danish pigs in the 1930s to 1950s showed reactions to several *Leptospira* serovars; i.e., Icterohemorrhagiae, Poi, Bataviae, Sejroe, Pomona and Saxkoebing [67]. A connection between reproductive problems and antibodies to the *Leptospira* serovars Bratislava and Muenchen in breeding pigs and in breeding herds has been observed [69,70]. In Finnish slaughter pigs, antibodies were found in 16.5% of (406) serum samples, reacting to one or more of the *Leptospira* serovars Hyos, Bataviae, Poi, Pomona, Sejroe and Icterohemorrhagiae [27]. A serological study was performed on farmed wild boars from 2005 to 2008: all 303 samples were negative for *Leptospira* spp. [71].

Serological investigations of pigs in Norway suggest that leptospirosis may occasionally be the cause of reproductive problems. In 1991, antibodies to *Leptospira* of the Australis serogroup were detected in two swine herds with clinical signs of reproductive disorders [72].

Information about leptospirosis in horses in the other Nordic countries is rather scarce. In Denmark, serological investigations have found antibodies in horse sera to *Leptospira* serovars Icterohemorrhagiae, Canicola, Grippotyphosa, Sejroe and Saxkoebing [67]. In Finland, horse sera collected at slaughterhouses showed that 32.6% (of 95) were positive for one or more of the serovars Bataviae, Poi, Sejroe, Icterohemorrhagiae and Hyos [27].

To the best of our knowledge, there are no published reports of leptospirosis in animals from Iceland.

#### 3.2.10. Surveillance of Leptospirosis in Animals in Denmark, Norway, Finland and Iceland

Leptospirosis in animals is notifiable in all Nordic countries, but the notification systems vary.

In Denmark, leptospirosis in cattle and swine is notifiable; i.e., a disease for which the farmers are obliged to call for a veterinarian and, if confirmed, a report has to be made to the Danish Veterinary and Food Administration (www.foedevarestyrelsen.dk, accessed on 1 May 2021).

Cattle and swine are tested before export depending on the requirements of the country exported to, but there is no active surveillance.

In Norway, leptospirosis in animals is a disease that should be reported to the Norwegian Food Safety Authority (https://www.mattilsynet.no/dyr_og_dyrehold/dyrehelse/dyresykdommer/bsykdommer.5294/binary/B-sykdommer, accessed on 15 May 2021).

No active or case-based surveillance is carried out. Analysis for *Leptospira* spp. is mainly undertaken upon clinical suspicion. In the last 10 years, about 140 samples have been analysed for *Leptospira* spp., most of them upon clinical suspicion. In one case, low antibody titres were found in 30 of 89 examined pigs but could not be confirmed as *Leptospira* infection. A few dogs (4/35) have been found positive for *Leptospira* spp. (personal communication).

In Finland, leptospirosis in animals is a notifiable disease but the notifications do not lead to any control measures (www.ruokavirasto.fi, accessed on 15 October 2022). Bulls used for artificial insemination (AI) are tested for *Leptospira*; otherwise, animals are mainly tested for leptospirosis upon clinical suspicion or before export.

In Iceland, leptospirosis is a notifiable disease in animals but there is no surveillance. There was a serological survey in 2004 of sows in Iceland, and a few of them were positive. However, there were no signs of disease (personal communication).

### 3.3. Treatment and Vaccinations

In both humans and animals, leptospirosis is treated with antibiotics, such as penicillin and doxycycline. The choice of antibiotic, administration regime, etc., differs depending on the severity and duration of symptoms. Early treatment may shorten the duration and the severity of the disease and prevent shedding of leptospires in the urine. Severe cases require hospitalization. The subject has been thoroughly covered by others in articles and by health organisations [5,7,13,17].

In general, vaccination of humans is restricted to persons in high-risk, endemic geographical regions and to individuals at risk through occupational activities. In the Nordic countries and most of Europe, vaccination of humans is not undertaken. One exception is France, where the Institute Pasteur developed a vaccine for Parisian sewer workers in the 1960s and 1970s [17]. For animals, at the global level, vaccines are produced and applied depending on the disease situation [13,16]. In Sweden, vaccination of dogs against leptospirosis is nowadays recommended for dogs travelling outside the country and/or visiting areas with known or suspected exposure to Leptospirosis. The vaccine used is a quadrivalent vaccine against four serogroups: Canicola, Icterohemorrhagiae, Australis, and Grippotyphosa (https://www.sva.se/amnesomraden/djursjukdomar-a-o/leptospiros-hos-hund/, accessed on 26 June 2023 [51]). An expert panel gathered by the International Society of Companion Animal Infectious Diseases (ISCAID) has recently prepared excellent guidelines and European consensus statements providing details about both treatment and vaccination of dogs (https://www.iscaid.org/guidelines/, accessed on 26 June 2023). 

## 4. Conclusions

The first human cases with leptospirosis in Sweden were diagnosed early in the 1900s. The incidence peaked in the 1930s and 1940s, followed by a sudden drop around 1950. Since then, the number of human cases has been low, and today only a few cases are reported every year. Studies of animals during the human peak period have shown that *Leptospira* spp. were frequently found in rats and occasionally in other animals and domestic animals. Serological surveys performed in the last 40–50 years have shown that Swedish animals are sometimes exposed to these bacteria but, nowadays, leptospirosis as a clinical disease is also regarded as (relatively) uncommon in animals.

The reason for this decrease in reported numbers of human and animal cases could be underreporting but also a true decline in cases. The diagnosis of leptospirosis is complicated and usually only performed at specific laboratories. Although the use of new molecular techniques has improved laboratory capacities, it is still a rather unique diagnosis. Additionally, clinical signs, both in humans and animals, can be very mild and nonspecific and not warrant any further investigation.

Other explanations could be better hygiene, better housing and better sanitary conditions.

Also, rodent control with more preventive measures and, generally, less contact with rodents and farm animals could be other explanations for the decrease in the number of human leptospirosis cases. Better animal hygiene and disease control are other tools for supressing the disease.

Since 2013, there has been a trend of human leptospirosis increasing in Europe, which has not been observed in Sweden or the other Nordic countries [3]. However, there is a risk that leptospirosis is a neglected disease in Sweden, and it deserves more attention now and in the future. Large outbreaks associated with heavy rainfall have been described in several countries, most of them in tropical areas [73]. However, this also happens in more temperate countries, as became evident in Denmark in 2011 when many people became ill after exposure to sewage in a flooded area in Copenhagen [74], and it will most likely occur more often in the future due to climate change. Flooding is not the only important risk for leptospirosis, and increased travelling to the tropics could also result in more imported cases that need attention and proper treatment. We suggest active surveillance including humans and domestic and wild animals. Stringent rodent control in society and animal farms is of outmost importance for prevention.

Surveillance of infections and prevention and containment of outbreaks caused by pathogens with a complex ecology and epidemiology, such as leptospirosis, require a One Health approach. The veterinary, public health and environmental authorities need to co-operate in strengthening these activities. Raising awareness within the medical and veterinary professions, as well as among the public, is pivotal.

## Figures and Tables

**Table 1:** Laboratory techniques that have been used or are currently in use in Sweden for diagnosis of leptospirosis in animals and humans.

Type of Technique	Type of Sample	Remarks
Culture	Clinical samples (e.g., urine, blood, cerebrospinal fluid (CSF)) and post mortem samples	Specific but slow, weeks to months due to slow growth of leptospires. Cumbersome, requires special culture media and skilled personnel
Animal inoculation	Clinical and post mortem samples	Specific but difficult, requires laboratory animals for inoculation. Regarded as unethical and not used today
Dark-field microscopy	Body fluids (e.g., urine, blood, CSF)	Quick but unreliable, specificity and sensitivity can be enhanced by combining with specific staining methods. Requires dark-field microscope
Levaditi stain	Post mortem or tissue samples	Slow, somewhat unspecific
MAT	Blood serum from humans and animals	Reference method (gold standard). Only performed at reference/expert laboratories Sensitive and specific, labour-intensive and complicated procedure
IgM-ELISA	Human blood serum	A commercial test: Serion ELISA classic IgM ESR125M (Institut Virion\Serion GmbH, Wurzburg, Germany). Simple and rapid assay, which is used today at the Public Health Agency of Sweden, Solna, Sweden
L. Hardjo ELISA	Bovine blood serum and (bulk) milk samples	An indirect ELISA, PrioCHECK^®^ L. Hardjo Ab Strip Kit, Prionics, Thermo Fisher Scientific, Lelystad, The Netherlands
PCR (molecular methods)	Blood, urine, CSF, post mortem samples	Sensitive, specific and quick. Requires special equipment and expertise for performing molecular laboratory diagnostics
Macroscopic slide agglutination test	Human blood serum	A simple, rapid test from the Institut Pasteur, Paris, France, and later from Bio-Rad, Berkeley, CA, USA. The test is no longer used at the Public Health Agency of Sweden

In Sweden, samples are analysed (almost) exclusively at the national reference laboratories of the Public Health Agency of Sweden (human specimens) and National Veterinary Institute (animal samples) with biosafety level two.

**Table 4:** *Leptospira* serovar distribution in dogs and horses in Sweden in 2007–2019. Sera tested with the MAT, and the threshold for positivity was a titre ≥ 1:100. Data from the archives of the National Veterinary Institute, Uppsala, Sweden.

*Leptospira* Serovar	Dogs	Horses
Australis	2	nt
Autumnalis	3	nt
Bratislava	17	7
Canicola	4	nt
Grippotyphosa	1	4
Icterohaemorrhagiae	82	5
Mus 2A (Sejroe)	77	3
Pomona	1	nt
Saxkoebing	8	nt
Total	195	19

nt = not tested for this serovar.

## Data Availability

All the relevant data are included in the manuscript in aggregated format.

## References

[1] Costa F., Hagan J.E., Calcagno J., Kane M., Torgerson P., Martinez-Silveira M.S., Stein C., Abela-Ridder B., Ko A.I. (2015). Global morbidity and mortality of leptospirosis: A systematic review. PLoS Neglected Trop. Dis..

[2] Pappas G., Papadimitriou P., Siozopoulou V., Christou L., Akritidis N. (2008). The globalization of leptospirosis: Worldwide incidence trends. Int. J. Infect. Dis..

[3] ECDC ECDC Surveillance Atlas of Infectious Diseases. https://atlas.ecdc.europa.eu/public/index.aspx.

[4] Adler B. (2015). History of leptospirosis and leptospira. Current Topics in Microbiology and Immunology.

[5] World Health Organization (WHO) (2003). Human Leptospirosis: Guidance for Diagnosis, Surveillance and Control.

[6] Picardeau M. (2017). Virulence of the zoonotic agent of leptospirosis: Still terra incognita?. Nat. Rev. Microbiol..

[7] Levett P.N. (2001). Leptospirosis. Clin. Microbiol. Rev..

[8] Vincent A.T., Schiettekatte O., Goarant C., Neela V.K., Bernet E., Thibeaux R., Ismail N., Mohd Khalid M.K.N., Amran F., Masuzawa T. (2019). Revisiting the taxonomy and evolution of pathogenicity of the genus Leptospira through the prism of genomics. PLoS Negl. Trop. Dis..

[9] Levett P.N. (2015). Systematics of leptospiraceae. Curr. Top. Microbiol. Immunol..

[10] Hartskeerl R.A., Terpstra W.J. (1996). Leptospirosis in wild animals. Vet. Q..

[11] Fennestad K.L., Borg-Petersen C. (1972). Leptospirosis in Danish wild mammals. J. Wildl. Dis..

[12] Dutta T.K., Christopher M. (2005). Leptospirosis—An overview. J. Assoc. Physicians India.

[13] Ellis W.A. (2015). Animal leptospirosis. Curr. Top. Microbiol. Immunol..

[14] Linglöf T. (1993). Opublicerad Intern Sammanställning på Statens Bakteriologiska Laboratorium.

[15] Faine S. (1982). Guidelines for the Control of Leptospirosis.

[16] World Organization for Animal Health (WOAH) (2022). Chapter 3.1.12, Leptospirosis. In Manual of Diagnostic Tests and Vaccines for Terrestrial Animals. https://www.woah.org/fileadmin/Home/eng/Health_standards/tahm/A_summry.htm.

[17] Haake D.A., Levett P.N. (2015). Leptospirosis in humans. Curr. Top. Microbiol. Immunol..

[18] Malmgren B. (1936). Om Weils sjukdom och dess förekomst i Sverige. Nord. Med. Tidskr..

[19] Olin G. (1933). Spirochaeta ictero-haemorrhagiae påvisad hos råttor i Sverige. Nord. Hyg. Tidskr..

[20] Olin G. (1934). Om Weils sjukdoms etiologi och epidemiologi med särskild hänsyn till svenska förhållanden. Sv. Läkaresällskapets Förhandlingar.

[21] Malmgren B. (1941). Studien über die Weilsche krankheit in Schweden. Acta Pathol. Microbiol. Scandinavica. Suppl..

[22] Nordström G. (1941). Weils sjukdom hos husdjuren. Nord. Med. Tidskr..

[23] Bourhy P., Bremont S., Zinini F., Giry C., Picardeau M. (2011). Comparison of real-time PCR assays for detection of pathogenic Leptospira spp. in blood and identification of variations in target sequences. J. Clin. Microbiol..

[24] Lau C.L., Smythe L.D., Craig S.B., Weinstein P. (2010). Climate change, flooding, urbanisation and leptospirosis: Fuelling the fire?. Trans. R. Soc. Trop. Med. Hyg..

[25] Lapidus H., Flaum A. (1923). Ett fall av Morbus Weil (Spirochaetosis ictero-haemorragica). Hygiea.

[26] Weil A. (1886). Über eine eigentümliche, mit Milztumour. Icterus und Nephritis einhergehende akute Infectionskrankheit. Dtsch. Arch. f. Klin. Med..

[27] Salminen A. (1956). Studies on the occurrence of various leptospiral types in Finland. Ann. Med. Exper. Biol. Fenniae.

[28] Borgen L.O., Thjøtta T.H. (1941). Weil’s Disease in Norway and the Occurence of Leptospira in Rats and Water.

[29] Koulumies R., Salminen A. (1953). Über leptospirosis in Finnland. Ann. Med. Intern. Fenniae. Suppl..

[30] Goris M.G., Boer K.R., Duarte T.A., Kliffen S.J., Hartskeerl R.A. (2013). Human leptospirosis trends, the Netherlands, 1925–2008. Emerg. Infect. Dis..

[31] Eves C., Kjelsø C., Benedetti G., Jørgensen C.S., Krogfelt K.A. (2023). Trends in human leptospirosis in Denmark, 2012–2021. Front. Cell. Infect. Microbiol..

[32] Holk K., Nielsen S.V., Rønne T. (2000). Human leptospirosis in Denmark 1970–1996: An epidemiological and clinical study. Scand. J. Infect. Dis..

[33] van Alphen L.B., Lemcke Kunoe A., Ceper T., Kähler J., Kjelsø C., Ethelberg S., Krogfelt K.A. (2015). Trends in Human Leptospirosis in Denmark, 1980 to 2012. Eurosurveillance.

[34] Arvidsson S., Hallgren J., Linder B., Snygg A.C. (1972). Leptospirosis—A topical case of Weil’s disease. Lakartidningen.

[35] SFS 2004:168 with the Amendments of SFS 2022:2017; Swedish Code of Statutes. https://svenskforfattningssamling.se/english.html.

[36] World Organization for Animal Health (WOAH) Old Classification of Diseases Notifiable to the OIE—List B. https://www.woah.org/en/what-we-do/animal-health-and-welfare/animal-diseases/old-classification-of-diseases-notifiable-to-the-oie-list-b/.

[37] National Veterinary Institute (SVA) (2021). Surveillance of Infectious Diseases in Animals and Humans in Sweden 2021.

[38] Backhans A., Jacobson M., Hansson I., Lebbad M., Lambertz S.T., Gammelgård E., Saager M., Akande O., Fellström C. (2013). Occurrence of pathogens in wild rodents caught on Swedish pig and chicken farms. Epidemiol. Infect..

[39] Strand T.M., Löhmus M., Persson Vinnersten T., Råsbäck T., Sundström K., Bergström T., Lundkvist Å. (2015). Highly Pathogenic Leptospira Found in Urban Brown Rats (Rattus norvegicus) in the Largest Cities of Sweden. Vector Borne Zoonotic Dis..

[40] Strand T.M., Pineda S., Backhans A., Jakobsen F., Råsbäck T., Lõhmus M., Järhult J.D., Lundkvist Å. (2019). Detection of Leptospira in Urban Swedish Rats: Pest Control Interventions as a Promising Source of Rats Used for Surveillance. Vector Borne Zoonotic Dis..

[41] Rubarth S. (1937). Weils sjukdom hos hund och räv. Medd. Från Sällskapet För Veterinärmedicinsk Forsk..

[42] Wendt J.V. (1956). Serological examinations for leptospirosis among domestic animals in Sweden. Nord. Vet. Med..

[43] Sandstedt K., Engvall A. (1985). Serum antibodies to Leptospira bratislava in Swedish pigs and horses. Nord. Vet. Med..

[44] Swedberg C., Eliasson-Selling L. (2006). Leptospira interrogans serovar bratislava hos gris-ett problem i Sverige?. Sven. Vet..

[45] Baverud V., Gunnarsson A., Engvall E.O., Franzén P., Egenvall A. (2009). Leptospira seroprevalence and associations between seropositivity, clinical disease and host factors in horses. Acta Vet. Scand..

[46] Akerstedt J., Lillehaug A., Larsen I.L., Eide N.E., Arnemo J.M., Handeland K. (2010). Serosurvey for canine distemper virus, canine adenovirus, Leptospira interrogans, and Toxoplasma gondii in free-ranging canids in Scandinavia and Svalbard. J. Wildl. Dis..

[47] Lundin T., Edlund A., Olsson Engvall E., Sandstedt K., Dotevall L. (2010). lnhemsk leptospiros hos hund i Sverige (fallbeskrivning). Sven. Veterinärtidning.

[48] Lindahl E., Boqvist S., Artursson K., Magnusson U. (2011). A field-study on Leptospira seroprevalence in dairy cows in four geographical areas in Sweden. Acta Vet. Scand..

[49] Boqvist S., Eliasson-Selling L., Bergström K., Magnusson U. (2012). The association between rainfall and seropositivity to Leptospira in outdoor reared pigs. Vet. J..

[50] Boqvist S., Bergström K., Magnusson U. (2012). Prevalence of antibody to six Leptospira servovars in Swedish wild boars. J. Wildl. Dis..

[51] Scahill K., Windahl U., Boqvist S., Pelander L. (2022). Leptospira seroprevalence and associated risk factors in healthy Swedish dogs. BMC Vet. Res..

[52] Gunnarsson A., Hurvell B., Hanko E. (1973). Isolering av Leptospira canicola från hund (A case report on the isolation of Leptospira canicola from dog). Nord. Vet. Med..

[53] Ellis W.A., McParland P.J., Bryson D.G., McNulty M.S. (1985). Leptospires in pig urogenital tracts and fetuses. Vet. Rec..

[54] Hathaway S.C., Little T.W. (1981). Prevalence and clinical significance of leptospiral antibodies in pigs in England. Vet. Rec..

[55] *SJVFS 2021:10*; Swedish Board of Agriculture’s Code of Statutes. https://jordbruksverket.se/om-jordbruksverket/forfattningar/.

[56] Krøjgaard L.H., Villumsen S., Markussen M.D., Jensen J.S., Leirs H., Heiberg A.C. (2009). High prevalence of *Leptospira* spp. in sewer rats (*Rattus norvegicus*). Epidemiol. Infect..

[57] Ottosen H.E. (1941). Om Leptospirainfektion hos rotter. Maanedsskr. Dyrlaeg..

[58] Rislakki V., Salminen A. (1955). Investigation of leptospirosis in rats in Finland. Acta Path Microbiol. Scand..

[59] Jensen P.M., Magnussen E. (2016). Is it too cold for Leptospira interrrogans transmission on the Faroese Islands?. Infect. Dis..

[60] Holm H. (1946). Leptospirosens klinik. Maanedsskr. Dyrlaeg..

[61] Ottosen H.E. (1962). Canine Leptospirosis in Denmark. J. Small Anim. Pract..

[62] Borg-Petersen C., Fennestad K.L. (1962). Incidence of canine leptospirosis in Denmark. Nord. Veterinärmedicin.

[63] Strande A. (1947). Leptospirose. Nor. Veterinaertidskrift.

[64] Sunde M., Heiene R., Fonnum K.J., Wold A. (2003). Leptospirose-en infeksjon med ny aktualitet?. Nor. Veterinærtidsskrift.

[65] Järvinen A.-K., Happonen I., Uusitalo M. (1986). Koiran leptospiroosi: Yleiskatsaus ja tapausselostus. Canine leptospirosis: A review and a case report. Soumen Eläinlääkärilehti.

[66] Borg-Petersen C., Fennestad K.L. (1956). Studies on bovine leptospirosis and abortion. I. Serological examination of aborting and “normal” cattle in Denmark. Nord. Veterinärmedicin.

[67] Fennestad K.L. (1956). Om leptospiroser hos vore husdyr. Nord. Veterinärmedicin.

[68] Schildt R., Laine M., Eskelinen E. (1987). Incidence of Leptospira antibodies in Finnish cattle. Suom. Elaeinlaeaekaerilehti.

[69] Jensen P.M., Binder M. (1989). Seroreaktion for leptospirose og reproduktionsproblemer hos danske svin. Er der en sammenhang? (Relationship between seroreaction to leptospires and reproduction failure in Danish swine?). Dan. VetTidsskr.

[70] Mousing J., Christensen J., Haugegaard J., Schirmer A.L., Friis N.F. (1995). A seroepidemiological survey of *Leptospira bratislava* infections in Danish sow herds. Prev. Vet. Med..

[71] Hälli O., Ala-Kurikka E., Nokireki T., Skrzypczak T., Raunio-Saarnisto M., Peltoniemi O.A., Heinonen M. (2012). Prevalence of and risk factors associated with viral and bacterial pathogens in farmed European wild boar. Vet. J..

[72] Saxegaard F., Ödegaard Ö. (1996). Leptospirose. Nor. Veterinärtidskrift.

[73] Bierque E., Thibeaux R., Girault D., Soupé-Gilbert M.E., Goarant C. (2020). A systematic review of Leptospira in water and soil environments. PLoS ONE.

[74] Wójcik O.P., Holt J., Kjerulf A., Müller L., Ethelberg S., Mølbak K. (2013). Personal protective equipment, hygiene behaviours and occupational risk of illness after July 2011 flood in Copenhagen, Denmark. Epidemiol. Infect..

